# Negotiating prices of drugs for rare diseases

**DOI:** 10.2471/BLT.15.163519

**Published:** 2016-08-31

**Authors:** Séverine Henrard, Francis Arickx

**Affiliations:** a Institute of Health and Society (IRSS), Université Catholique de Louvain, Clos Chapelle-aux-Champs, 30 B1.30.15, 1200 Brussels, Belgium.; b National Institute for Health and Disability Insurance (RIZIV/INAMI), Brussels, Belgium.

Rare diseases are often serious, chronic and life-threatening. The European Union (EU) definition of a rare disease is one that affects fewer than 5 in 10 000 people.^1^ At present, more than 6000 rare diseases are known; around 80% of them are genetic disorders and half of them occur during childhood. Grouped together, rare diseases affect 6–8% (or about 30 million people) out of the 508 million population of EU countries.^2^ This roughly equals the estimated prevalence of diabetes in the World Health Organization European Region, which in 2013 was 6.8% of 658.7 million adults in the 20–79 year age group.^3^ This illustrates the paradox of rarity: each disease is rare but collectively, rare diseases affect many people.^4^ In 2009, responding to increasing concerns about the issue, the European Council published a recommendation (2009/C 151/02) on action in the field of rare diseases, recommending Member States to adopt and establish national plans for rare diseases by the end of 2013.^2^

Rare diseases are associated with a high psychological burden for the patient but they can also have a major impact on a patient’s family.^5^ In addition to the health burden on patients, few of these diseases have an effective drug treatment available. This is because the drugs to treat rare diseases (called orphan drugs) are not commercially viable for pharmaceutical companies, due to the small number of patients per disease. To encourage pharmaceutical companies to develop and market orphan drugs, the European Parliament and the European Council published the regulation (EC) No 141/2000 on orphan medicinal products in 1999.^1^ Orphan designation for a medicine is granted at the European level by the European Medicines Agency (EMA). Three criteria must be met, as stated in the regulation: “(i) the medicine must be intended for the treatment, prevention or diagnosis of a disease that is life-threatening or chronically debilitating; (ii) the prevalence of the condition in the European Union must be no more than 5 in 10 000 or it must be unlikely that the marketing of the medicine would generate sufficient returns to justify the investment needed for its development; and (iii) no satisfactory method of diagnosis, prevention or treatment of the condition concerned can be authorized, or, if such a method exists, the medicine must be of significant benefit to those affected by the condition”.^1^ The application for orphan designation, which can be submitted by the sponsor of the medicinal product at any stage of drug development, is then examined within 90 days by the Committee for Orphan Medicinal Products of the EMA.

When a medicine is granted orphan designation, several incentives are provided to the sponsor of a clinical trial, as stated in regulation (EC) No 141/2000.^1^ These incentives include protocol assistance, scientific advice from EMA before the submission for marketing authorization and market exclusivity of 10 years once the medicine is marketed. This period of market exclusivity can be extended by two years for medicines that comply with an agreed investigation plan for medicines for paediatric care. Finally, depending on the status of the sponsor and the type of service required, reductions in fees to be paid for regulatory activities related to the evaluation of orphan medicinal products by EMA (e.g. protocol assistance, application for marketing authorization) can also be available for the sponsor.

**Fig. 1:**
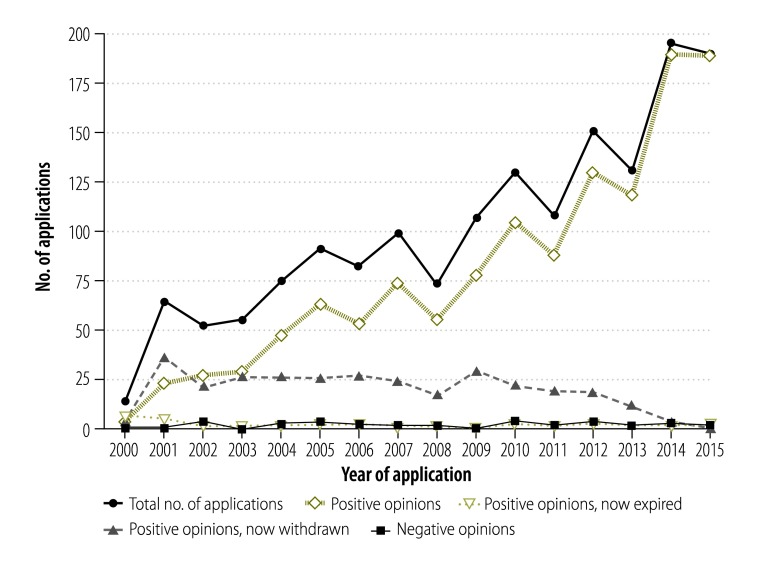
Number of applications for orphan designation for medicinal products to the European Medicines Agency over the years 2000–2015

Notes: Positive opinions: number of medicinal products that were granted orphan designation and that still have this designation granted. Positive opinions, now expired: number of medicinal products that were granted orphan designation, but that are now removed from the register as the 10-year market exclusivity after the market authorization has expired. Positive opinions, now withdrawn: number of medicinal products that were granted orphan designation, but that were later withdrawn. Negative opinions: number of medicinal products that were not granted orphan designation.

By the end of 2015, 89 different orphan medicinal products had received authorization to enter the market from the EMA’s Committee for Medicinal Products for Human Use, and 123 other medicinal products intended for rare diseases but without the orphan designation in Europe also received the European marketing authorization.^6^ In addition, an increasing number of applications for orphan designation are submitted to EMA each year (Fig. 1).^7^ As a consequence, an increasing number of applications for a marketing authorization of an orphan medicinal product is observed each year. In 2015, about one-fifth (18 out of 93) of the new medicines recommended by the EMA for marketing authorization had an orphan designation. The International Rare Diseases Research Consortium is also working in this area. Set up in April 2011 by the European Commission and the United States National Institutes of Health, the consortium comprises researchers and organizations investing and collaborating in research for rare diseases. Its goal is to contribute to the development of 200 new medicinal products for rare diseases in the United States of America and Europe by 2020. Thanks to this mobilization, an increasing number of patients living with a rare disease have or can hope to have a treatment for their condition.

This success, however, comes at a price for health-care systems and hence for society. Only a small number of patients use each rare disease drug and therefore pharmaceutical companies compensate for the lower market size with a higher price for the product.^4^ For example, a study published in Belgium estimated the mean total lifetime cost of replacement therapy for haemophilia, a rare bleeding disorder, as euro (€) 6 million per incident case.^8^ A study published in 2011 showed that orphan-designated drugs were associated with a higher median price than non-orphan-designated drugs for rare diseases.^9^ The median price per defined daily dose was €138.56 for 28 orphan drugs and €16.55 for 16 comparable non-designated orphan drugs.^9^

Due to the increasing number of treatments for rare diseases that have been approved and are under development, and hence the rising costs for countries’ health systems, there is an ongoing debate about health policies for rare diseases and particularly about reimbursement of rare disease drugs.^4^^,^^10^^,^^11^ Currently, when marketing authorization is granted for a medicine by EMA, the pricing and reimbursement is negotiated individually with pharmaceutical companies in each European country, where national health policies vary. The pricing of a drug can be set by the manufacturers (e.g. for generics and non-reimbursable drugs), fixed, or negotiated with the pharmaceutical company, depending on the health-care system in the country. The drug’s pricing is set by pharmaceutical companies based on the costs of research and development, the projected number of patients who will benefit from the drug and a profit margin.^12^ Other factors are also taken into account to set the pricing of a drug, depending on the country: clinical performance; economic evaluation (cost–effectiveness); availability and cost of existing alternative treatments; international prices of the product; and the degree of innovation of the product.^12^^,^^13^ In Europe, the price of a drug can be controlled for all products, controlled for reimbursable pharmaceuticals only or not regulated, depending on the country. As a consequence, the availability, price and utilization of an orphan drug can differ markedly across different European countries. Decision-making about the availability, pricing and patient reimbursement of orphan drugs should be done according to clearly defined criteria.^11^^,^^14^

To resolve this issue, two European countries – Belgium and the Netherlands – have teamed up to negotiate the pricing of orphan drugs with pharmaceutical companies. An announcement was made by the health ministers of the two countries in April 2015 during the informal meeting of European Ministers for Employment, Social Policy, Health and Consumer Affaire in Riga, Latvia. A pilot project was scheduled to begin in 2016. Since the agreement was signed, several pharmaceutical companies have declared their willingness to cooperate in the pilot project. In September 2015, the Grand Duchy of Luxemburg joined the Belgium–Netherlands project and the health ministers of other EU countries have demonstrated their interest in the project. 

This collaboration has a potential triple benefit: (i) for health system sustainability; (ii) for pharmaceutical companies; and (iii) for patients having a rare disease. First, by combining the population of three countries in negotiations, the number of patients who will use the drug (i.e. the market size) will be higher. On this basis, health ministers could be in a better position to negotiate favourable pricing for the new drug. This could therefore help the sustainability of each country’s health system. Second, pharmaceutical companies have guarantees that their product will be delivered to a bigger market. They also gain by submitting only one reimbursement dossier for three countries rather than one in each country. Third, and, most importantly, patients benefit by getting access to the treatment and possibly faster access than otherwise. Beyond the negotiation of pricing for drugs for rare diseases, the three countries involved announced they will also exchange information, share patient registers and coordinate evaluation methods (i.e. health technology assessment) for rare diseases. This is important, as it will allow the market size of each rare disease to be estimated for potential manufacturers, while lowering drug pricing.

In conclusion, up to now the high cost of drugs for rare diseases has been affordable for health-care systems in the EU, given the low number of patients benefitting from the existing marketed drugs. However, the increasing number of new orphan drugs marketed every year could start to threaten the sustainability of health-care systems in the EU. We highlight here the importance of organizing a debate among EU countries on negotiations about the pricing of medicines for rare diseases, and teaming up to achieve this. This joint initiative of the health ministries of Belgium and the Netherlands, and now Luxembourg, is a promising start.
